# An MRI study of solute transport in the intervertebral disc

**DOI:** 10.1007/s10334-019-00781-z

**Published:** 2019-10-04

**Authors:** Rachel M. Palfrey, Ian R. Summers, C. Peter Winlove

**Affiliations:** 1 Medical Imaging, University of Exeter, Exeter, EX1 2LU UK; 2 Physics and Astronomy, University of Exeter, Exeter, EX4 4QL UK

**Keywords:** Intervertebral disc, Microcirculation, Contrast agent

## Abstract

**Objective:**

Quantitative magnetic resonance imaging was used to determine partition coefficients and characteristic time constants for diffusion of MRI contrast agents in disc tissue.

**Materials and methods:**

Twenty-two excised equine intervertebral discs were exposed to a range of contrast agents: six to manganese chloride, eight to Magnevist (gadopentetate dimeglumine) and eight to Gadovist (gadobutrol), and uptake into the disc was quantified in *T*_1_-weighted images.

**Results:**

Diffusion for all contrast agents was approximately 25% faster in the nucleus than in the outer annulus; disc-average time constants ranged from (2.28 ± 0.23) × 10^4^ s for Gadovist (uncharged, molecular mass 605 g/mol) to (5.07 ± 0.75) × 10^4^ s for the manganese cation (charge + 2). Disc-average partition coefficients ranged from 0.77 ± 0.04 for the anion in Magnevist (charge − 2, molecular mass 548 g/mol) to 5.14 ± 0.43 for the manganese cation.

**Conclusion:**

The MRI technique provides high-quality quantitative data which correspond well to theoretical predictions, allowing values for partition coefficient and time constant to be readily determined. These measurements provide information to underpin similar studies in vivo and may be used as a model for the transport of nutrients and pharmaceutical agents in the disc.

## Introduction

Seventy percent of the world’s population suffers from low back pain [1] and it imposes a costly burden in terms of health care and loss of productivity at work [2]. The aetiology of low back pain is still unclear but deterioration of the intervertebral disc is widely implicated [3]. By the age of 49, 97% of lumbar intervertebral discs show some degeneration [4]. Although there is difficulty in distinguishing between natural ageing and pathological disorders of the intervertebral disc [2], many authors (e.g., [1]) have expressed the hope of slowing the process of degeneration, or even reversing it, to benefit patients.

There is, therefore, an urgent need to identify and thence to diagnose the factors initiating disc degeneration. The intervertebral discs are the largest avascular structures in the body [5]. In humans, cells at the centre of the disc can be as far as 8 mm from the nearest blood vessel [5] and are reliant for their nutrition on transport of solutes through the extracellular matrix [6, 7]. Though a link has long been recognised between degeneration and a lack of nutrient supply to the disc [8, 9], it is not clear whether nutrient exchange is compromised by changes in the microcirculation surrounding the disc or by alterations in the solute permeability of the extracellular matrix of the disc itself. The mechanisms and factors determining the rate of transport are still not well understood [10, 11], in part due to technical difficulties in investigating solute transport in the disc in vivo.

Many years of experimental research on excised discs, augmented by theoretical modelling, have established that within the disc itself diffusion is the main transport mechanism for small solutes, with convection playing a more important role in the transport of larger solutes [12–14]. The sensitivity of transport rate to the size and charge of the solute [15, 16] and to matrix structure and composition have been extensively investigated [17, 18]. Results show that diffusivity tends to decrease with the molecular size of the solute and increase with tissue water content, the latter relating to pore size in the tissue; partition coefficient tends to decrease with molecular size and varies with water content and fixed charge density in the tissue.

In vivo, solutes which exchange between blood and disc cells encounter serial barriers in addition to the extracellular matrix of the disc itself: the end plate, subchondral bone and the walls of the blood vessels of the bone and outer annulus. Much work is still required to resolve their individual effects on transport. This will ultimately require measurements in vivo on the intact spine, and MRI is likely to be an important tool for this task. MRI has been widely used to evaluate intervertebral disc degeneration, infection and trauma, e.g., [19–21]. The uptake of contrast agents has also been investigated, using a comparison of pre- and post-contrast images, the latter taken after a delay [22]. Studies involving contrast agents have established correlations between the uptake rates of charged molecules and disc proteoglycan content, e.g., [23], or disc degeneration [24, 25], though with somewhat inconsistent results. These discrepancies arise in part because of differences in patient selection and disc characterisation, but a major variable is the MRI contrast agent employed. Contrast agents vary in charge, molecular size and molecular structure, all of which can influence their interactions with each of the barriers they encounter in passing from blood into disc tissue.

The present research is an in-vitro study, designed to inform the development of MRI for use as an in-vivo quantitative probe of solute exchange between blood and disc tissue. The uptake of representative MRI contrast agents in disc tissue is compared, with measurement of characteristic time constants and partition coefficients at various locations in the disc nucleus and annulus. Equine caudal discs are used, which resemble in size and structure the normal, young human intervertebral disc. (The human intervertebral disc is approximately 7–10 mm thick and around 40 mm in diameter in the lumbar spine [2]. The equine caudal discs used in the present study have similar thickness (6–11 mm) and diameters in the range 19–28 mm. Along with a somewhat smaller diameter, the equine discs have correspondingly fewer lamellar rings in the annulus.) The in-vitro MRI technique allows continuous measurements over a period of 15–20 h, providing an understanding of contrast-agent movement that would be impracticable to obtain in vivo. Two of the three contrast agents investigated are suitable for use in human studies.

## Materials and methods

### Sample preparation

The investigation employed 22 intervertebral discs from the tails of 12 horses that had been euthanised for reasons unrelated to the research project. The animals were aged by their dentition (range 2–30 years). Fresh horse tails were transported from the abattoir (Potters, Taunton, UK) to the laboratory where the discs were excised. Experiments were either performed on the same day, or discs were frozen and thawed for experiments at a later date (depending on the availability of horses and imaging sessions). Measured diffusion characteristics do not suggest any systematic difference between fresh and frozen samples.

**Fig. 1:**
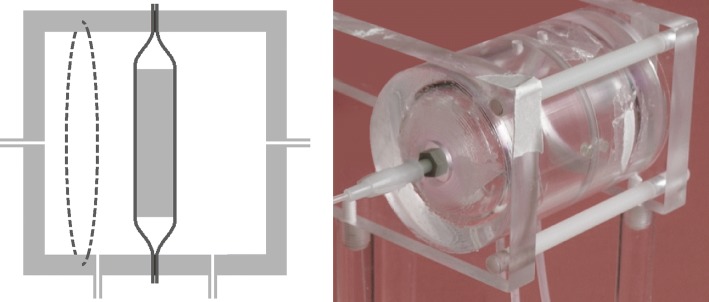
(left) Diagram showing the cylindrical incubation chamber with a disc supported centrally between two pieces of dialysis membrane; (right) photograph of the chamber itself. The design is adapted from Urban [26]; the internal diameter is 50 mm; drainage and filling tubes allow the liquid in the chamber to be changed while it is in the MRI scanner

A major problem in studying the permeability properties of the intervertebral disc in vitro is that the mechanically unconstrained disc swells in physiological media. This can be prevented by imposing an appropriate osmotic load on the tissue [26] which is achieved in the present study by means of a purpose-made incubation chamber (Fig. 1). Contact between the incubation medium and the dorsal and ventral surfaces of the disc is via two pieces of dialysis membrane (Medicell International, cut-off 12,000–14,000 g/mol) which impose the requisite osmotic pressure gradient. A quantity of 0.15 M NaCl, pH 7.4, fills the space immediately around the disc to maintain hydration and provides a transport pathway; the two compartments on either side of the membranes are filled with 0.15 M NaCl containing 10% w/v polyethylene glycol (PEG), molecular mass 20,000 g/mol. (Preliminary experiments established that this concentration of PEG maintained the initial hydration over several hours.) The disc was allowed to equilibrate in the chamber for at least 1 h before the start of the imaging experiment (see below); after some initial images the NaCl/PEG medium was exchanged for a similar medium to which an MRI contrast agent had been added.

### Imaging

Magnetic-resonance imaging was carried out with a 1.5 T scanner (Philips Intera), using a 45-mm-diameter microscopy coil. A cod-liver-oil capsule and a small phial of distilled water were attached to the incubation chamber when it was positioned in the scanner, allowing normalisation of signal intensity against scanner drift over a long series of dynamic scans. Typically, the greyscale values of the oil and water references changed by a few percent over a scan series. For each of the dynamic scans, correction factors were calculated from each reference according to the ratio of the reference intensity to the average reference intensity (average over all scans in the series); greyscale values were scaled using the mean of the two correction factors.

An initial high-resolution anatomical scan was performed (TE 11 ms, TR 33 ms, flip angle 30°, 20 contiguous slices, slice thickness 1 mm, FOV 60 × 60 mm, 576 × 576 pixels) to allow accurate location of the regions of interest (ROIs) within the disc (see below). This was followed by a single *T*_1_-weighted scan—details as in the next paragraph—to act as a baseline for the subsequent imaging. Then the NaCl/PEG medium was drained from both halves of the incubation chamber, and the chamber was refilled with medium to which one of three paramagnetic contrast agents had been added: manganese chloride, Magnevist (gadopentetate dimeglumine, Bayer PLC) or Gadovist (gadobutrol, Bayer PLC). Of the 22 disc samples, six were perfused with manganese chloride, eight with Magnevist and eight with Gadovist. (Magnevist and Gadovist are clinically approved; manganese chloride is unsuitable for use in humans because of its cellular toxicity).

**Fig. 2:**
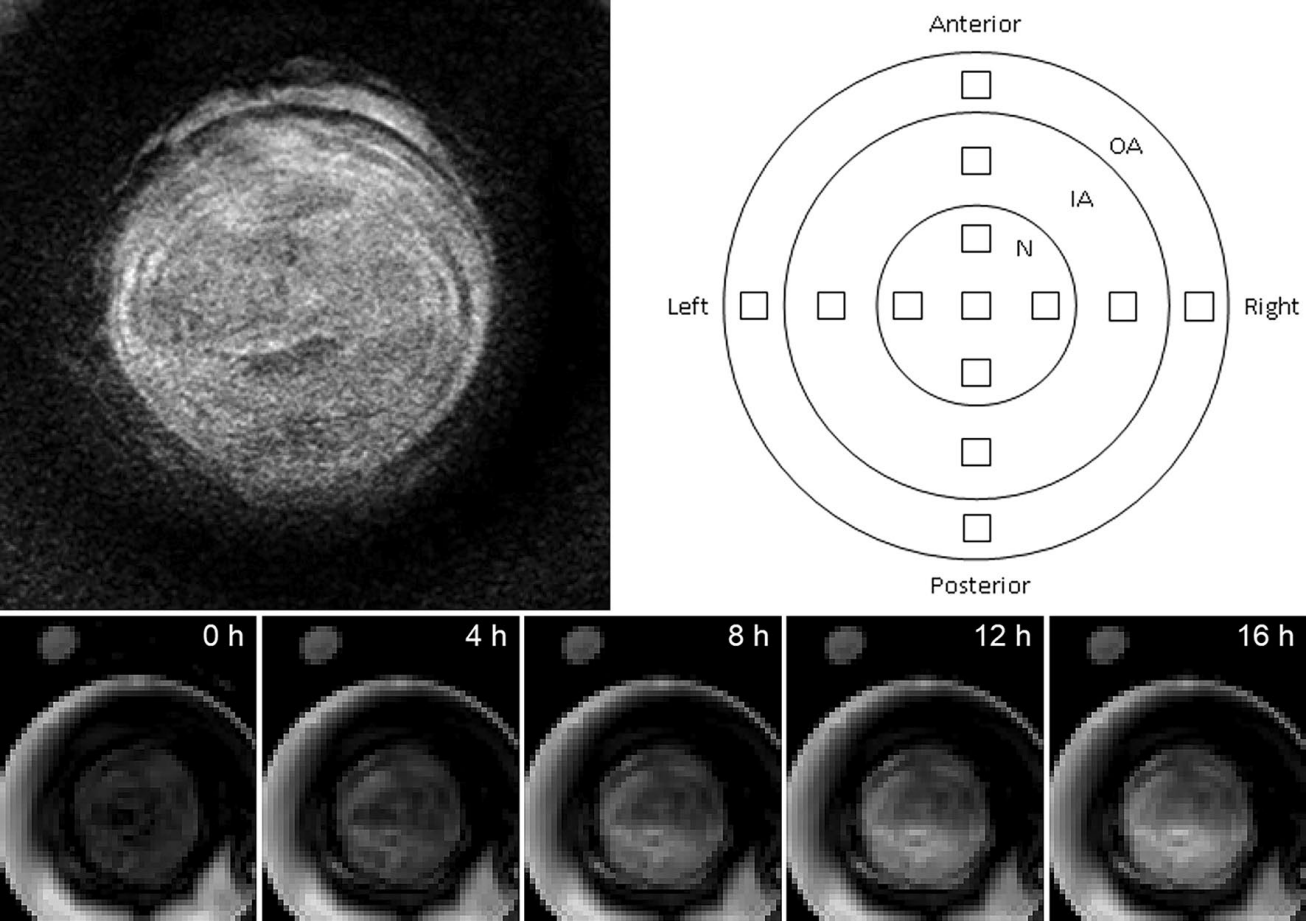
(top left) Central slice from an anatomical scan; (top right) schematic diagram of the 13 disc ROIs; N = nucleus, IA = inner annulus and OA = outer annulus; individual regions are labelled as inner left annulus = ILA, outer posterior annulus = OPA, etc. (the positions of the various regions have not been adjusted to match the anatomical scan); (bottom) central slices at 4-hour intervals from a series of dynamic scans for the diffusion of Gadovist, showing the disc in the centre of the incubation chamber and the oil reference outside; (the bright areas near the chamber walls show regions of incubation medium, where the image slice falls outside the dialysis membranes)

After the incubation chamber had been refilled, *T*_1_-weighted dynamic scans were repeated (approximately one every three minutes) for a period of several hours—average period 17 h, longer or shorter according to scanner availability. The parameters of each scan were: TE 3.9 ms, TR 20 ms, flip angle 30°, 30 slices, slice thickness 2 mm, slice overlap 1 mm, FOV 60 × 60 mm, 64 × 64 pixels, resampled to 576 × 576 pixels so as to match the anatomical scan. Example images, showing the time variation of greyscale values in the disc, are given in Fig. 2 (lower panels). The anatomical scan and the dynamic scans were pre-processed using the “CLEAR” function in the Philips imaging software, which corrects the image uniformity on the basis of a coil sensitivity map acquired in a reference scan.

The concentrations of contrast agent (manganese chloride 0.125 mM, Magnevist 1.8 mM and Gadovist 2.25 mM) were determined on the basis of preliminary experiments—high enough to produce measurable effects on the *T*_1_ relaxation time of the disc but not so high as to reduce the MRI signal because of changes to the *T*_2_ relaxation time.

The contrast agents were selected because of their differing charge, molecular size, and molecular structure to explore the effects on solute mobility of electrostatic interaction (predominantly with the negatively charged proteoglycans) and steric interaction (with both the proteoglycans and the collagen fibres in the extracellular matrix of the disc). The contrast-producing component of manganese chloride is the manganese cation with charge + 2; the contrast-producing component of Magnevist is the Gd-DTPA anion with charge − 2; Gadovist is non-ionic and hence has no net charge. The Gd-DTPA anion in Magnevist has a molecular mass of 548 g/mol; Gadovist has a molecular mass of 605 g/mol. Together they cover a range of sizes and charge similar to those of molecules involved in disc nutrition and of potential therapeutic agents, whose rates of transport into the disc are of interest.

**Fig. 3:**
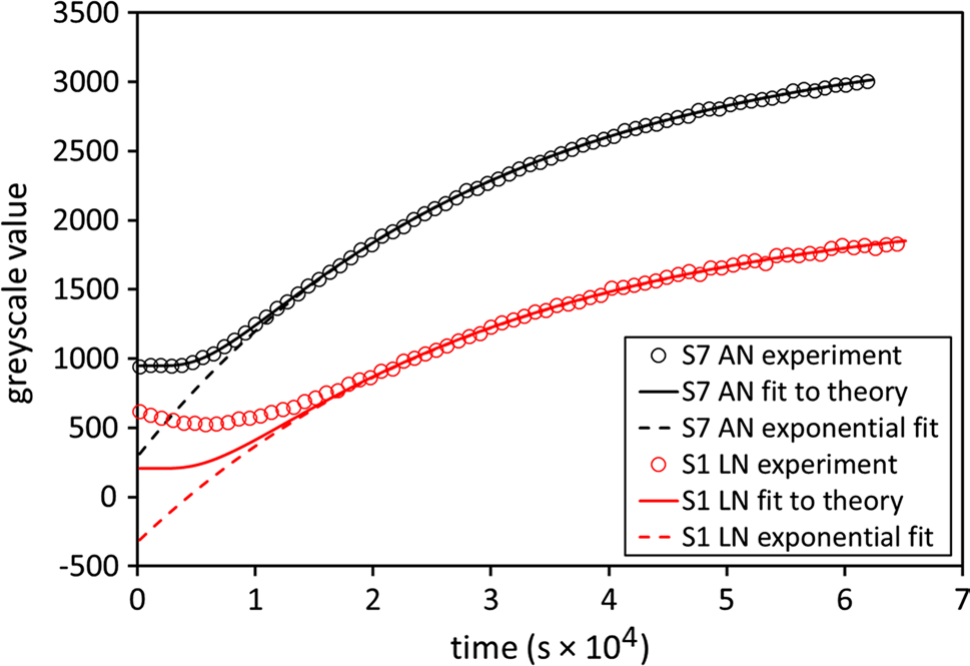
Typical greyscale time courses for two ROIs in samples perfused with manganese chloride. Increasing greyscale value corresponds to increasing image brightness, resulting from increasing concentration of contrast agent. Sample 7, anterior-nucleus (AN) region produces data which fit the expected theory (upper curves). Sample 1, left-nucleus (LN) region produces data which only fit the expected theory in the later part of the time course (lower curves). The r.m.s. discrepancy between the experimental data points and the curve fits (calculated for times greater than 2 × 10^4^ s) is around ten greyscale units in each of the cases shown. For clarity, only one in five of the experimental data points is plotted

The initial high-resolution anatomical scan of the disc (Fig. 2) allows the annulus, with its distinctive rings, to be distinguished from the nucleus. On the basis of this scan, 13 ROIs were positioned on the central axial slice of each dynamic scan, as shown in Fig. 2; each ROI has approximate volume 2 mm^3^ (1 mm^2^ in plane); positions were varied to correspond with the different dimensions of individual discs. For each ROI within each sample, data were obtained on the variation of greyscale values with time, reflecting the diffusion of the active component of the contrast agent into the disc. Examples are given in Fig. 3.

With hindsight, it is clear that the time resolution of the MRI data is much higher than necessary (note that Fig. 3 shows only one in five data points). It would be possible to obtain higher spatial resolution in the dynamic scans using less frequent scans of longer duration, with no significant loss of information about the time course.

### Data processing

According to the solution of the diffusion equation in one dimension [27], the time variation of the contrast-agent concentration *C* on the central axial plane of the disc is expected to be of the form:

1 $$ C\left(t\right)={C}_{\infty }\left[1-\frac{4}{\pi }\sum_{odd\,n}\frac{1}{n}{\left(-1\right)}^{\frac{n-1}{2}}\text{exp}\left(-{n}^{2}t/\tau \right)\right], $$

where *C*_*∞*_ is the concentration at long times and *τ* is a characteristic time constant. (Use of a one-dimensional analysis implies the assumption that diffusion from the faces of the disc is dominant over radial diffusion, inwards from the outer annulus).

At the contrast-agent concentrations encountered in these experiments, the MRI sequence used for the dynamic scans produces images whose greyscale values *G* are determined by concentration *C* according to an offset linear relationship [28]. Hence, the time course of greyscale values on the central axial slice is expected to follow an equation of the same form as (1), with the same time constant *τ*:

2 $$G\left(t\right)=A-B\sum_{odd\,n}\frac{1}{n}{\left(-1\right)}^{\frac{n-1}{2}}\mathrm{exp}\left(-{n}^{2}t/\tau \right),$$

where *A* and *B* are constants. For *t* ≥ *τ*/2, the higher order terms in the summation are negligible (less than 1% of the first-order term) and Eq. 2 reduces to

3 $$G\left(t\right)=A-B\,\mathrm{exp}\left(-t/\tau \right).$$

In practice, this expected behaviour was observed in many cases. Figure 3 shows two examples of the time course of greyscale values, for selected regions of samples perfused with manganese chloride. For the anterior-nucleus region of sample 7 (upper curves), the experimental data (circular markers) can be fitted with a curve of the form given by Eq. 2 (full line), as expected, and the later part of the time course can be fitted with an exponential of the form given by Eq. 3 (dashed line). However, in a number of cases the experimental data did not follow the theoretical prediction in the early part of the time course—greyscale values rose or fell in the initial phase, rather than remaining constant as predicted by Eq. 2. For example, for the left-nucleus region of sample 1 (lower curves in Fig. 3), only the later part of the experimental data (circular markers) can be fitted with a curve of the form given by Eq. 2 (full line). The later part of the time course can also be fitted with an exponential of the form given by Eq. 3 (dashed line). There is no obvious explanation for such departures from the predicted behavior—presumably they reflect changes to the disc, other than diffusion of the active component of the contrast agent. Only the later part of each time course was used for curve fitting, to avoid problems from these inconsistencies in the initial phase.

The experimental data for each region of each sample were fitted to an exponential of the form given by Eq. 3 except in a few cases, where the time constant *τ* was very long, so that the exponential part of the time course was not established before the end of the measurement period—in those cases the data were fitted to Eq. 2. (In a very small number of cases—two from 286—no convincing curve fit was possible.) Each curve fit gave values for the amplitude *B* and the time constant *τ*. Theory [27] predicts that *τ* is proportional to the square of the disc thickness—to compensate for any spread of results due to this effect, data for *τ* from individual discs (thickness *d* in the range 6–11 mm) were normalised to a disc thickness of 9 mm; time constants were multiplied by a correction factor of the form (*d*_0_/*d*)^2^, where *d*_0_ = 9 mm. The long-time concentration *C*_*∞*_ of contrast agent in a ROI can be expressed as a partition coefficient *C*_*∞*_/*C*_0_, where *C*_0_ is the concentration of contrast agent in the incubation medium. A value for partition coefficient was calculated from each value of amplitude *B* using the conversion:

4 $$\frac{{C}_{\infty }}{{C}_{0}}=\frac{\pi }{4}\left(\frac{B}{f\Delta G}\right),$$

where ∆*G* is the change in greyscale value of the incubation medium neighbouring the disc when liquid with no contrast agent (image from baseline scan) was replaced by liquid with contrast agent at concentration *C*_0_ (image from first dynamic scan), and *f* is the water fraction (by volume) of the disc; the constant (π/4) arises from comparison of Eqs. (1) and (2). The water fraction *f* was calculated on the basis of the disc’s mass (average of pre-incubation and post-incubation measurements) and its mass after subsequent desiccation, assuming a density of 1300 kg m^–3^ for the non-water component of the disc [29]. All data are available from the University of Exeter Repository.

## Results

**Fig. 4:**
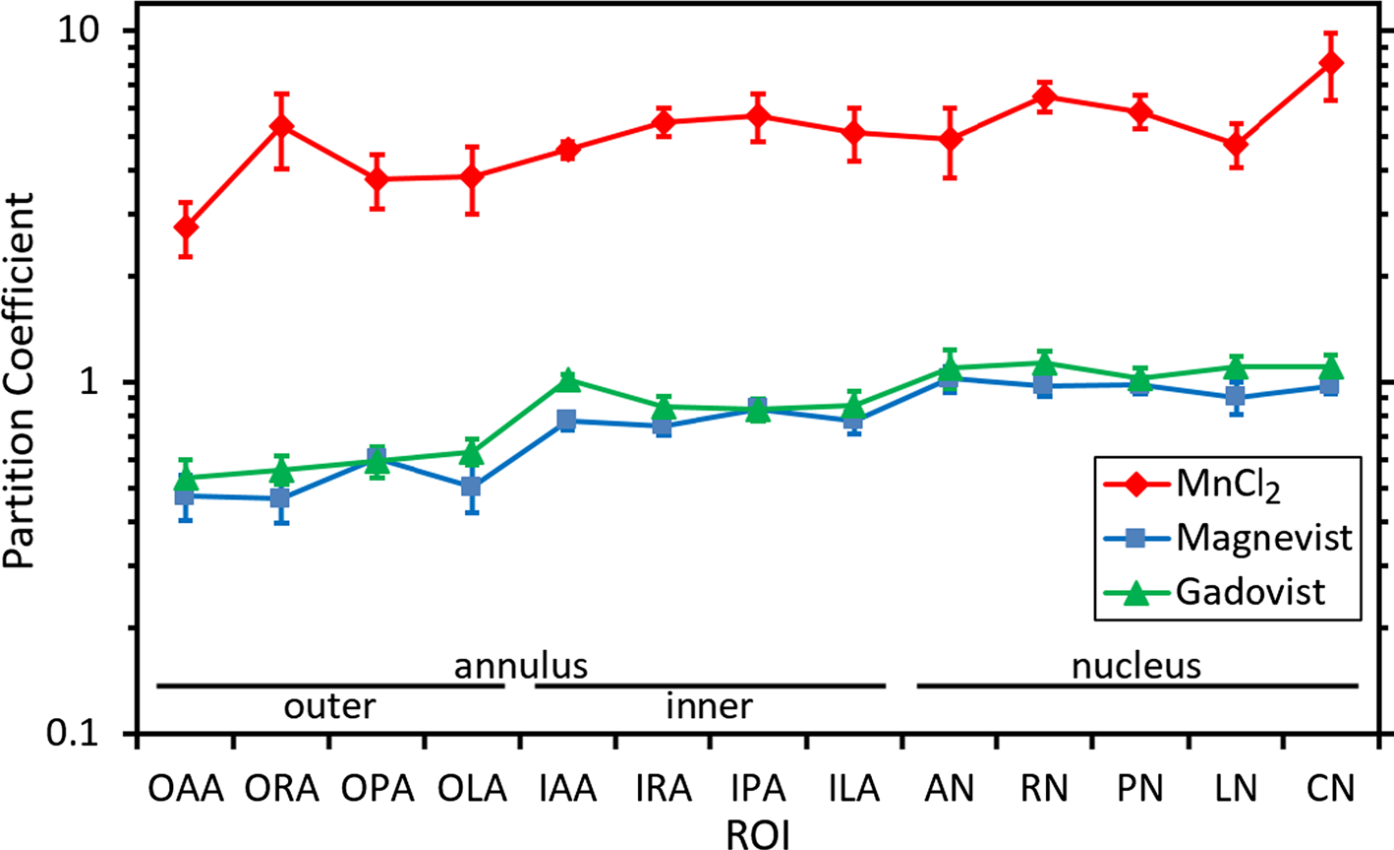
Mean values for the partition coefficient *C*_∞_/*C*_0_ for the different disc regions, with separate data for each of the contrast agents; the ROIs are labelled according to their position within the disc: outer annulus, inner annulus or nucleus (see Fig. 2); the error bars represent the standard error in the mean

Figure 4 shows mean values for the partition coefficient *C*_*∞*_/*C*_0_ for the different disc regions, with separate data for each of the contrast agents. For Magnevist and Gadovist the values of partition coefficient are mostly close to unity. However, higher values are observed for manganese chloride, i.e., in that case the concentration of the contrast-producing manganese ions within the disc is higher than the external concentration. Average values over all ROIs are 5.14 ± 0.43, 0.77 ± 0.04 and 0.87 ± 0.04, for manganese chloride, Magnevist and Gadovist, respectively, where the stated errors are calculated from the variability between discs. The difference between partition coefficients for manganese chloride and Magnevist is statistically significant (*p* < 0.001, Tukey test on log data); similarly, the difference between manganese chloride and Gadovist is significant (*p* < 0.001); the difference between Magnevist and Gadovist is not significant (*p* = 0.33).

**Fig. 5:**
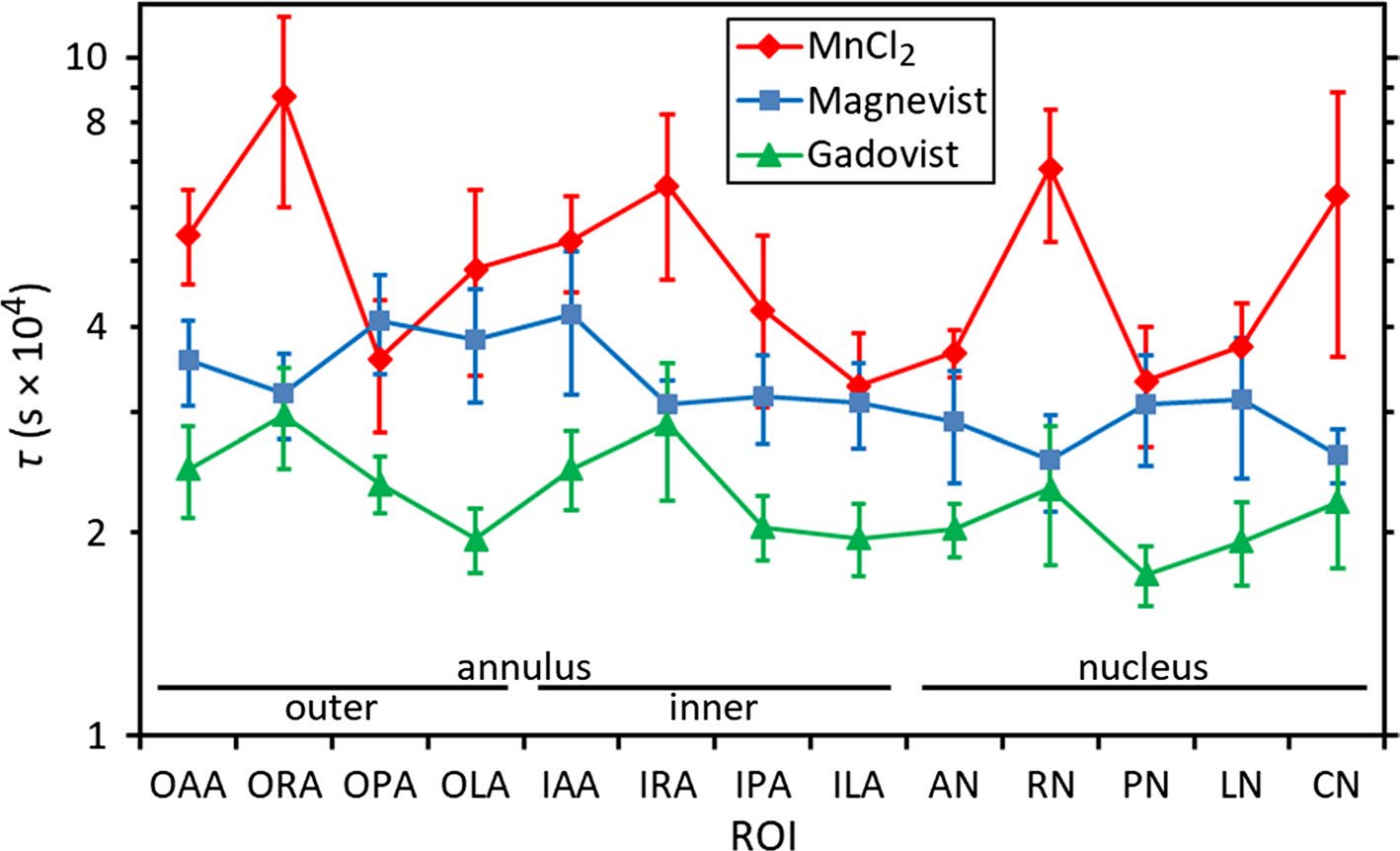
Mean values of the characteristic time constant *τ* for the different disc regions, with separate data for each of the three contrast agents; the ROIs are labelled according to their position within the disc: outer annulus, inner annulus or nucleus (see Fig. 2); the error bars represent the standard error in the mean

Figure 5 shows mean values of the characteristic time constant *τ* (normalized—see above) for the different disc regions, with separate data for each of the contrast agents. Average values over all ROIs are (5.07 ± 0.75) × 10^4^ s, (3.27 ± 0.34) × 10^4^ s and (2.28 ± 0.23) × 10^4^ s, for manganese chloride, Magnevist and Gadovist, respectively, where the stated errors are calculated from the variability between discs. In other words, time constants of approximately 14, 9 and 6 h are observed for diffusion over a distance of around 4 mm. The difference between time constants for manganese chloride and Gadovist is statistically significant (*p* < 0.001, Tukey test on log data); the differences between manganese chloride and Magnevist (*p* = 0.055) and Magnevist and Gadovist (*p* = 0.075) narrowly fail to reach significance at the *p* = 0.05 level.

## Discussion

As outlined previously, the active component of the manganese-chloride contrast agent is the positively charged manganese ion. The high values of partition coefficient that are observed for manganese chloride (Fig. 4), i.e., significantly greater than one, can be attributed to the presence of proteoglycans in the disc [30] which are associated with fixed negative charge and thus, because of the requirement for electroneutrality, are also associated with a raised concentration of cations in the interstitial fluid of the disc. Diffusion of manganese ions into the disc is enhanced by the pool of cations to which they have access. This effect does not apply in the case of Gadovist, since it is non-ionic: as expected, the observed values of partition coefficient (Fig. 4) are close to one in that case.

By a similar argument, it might be expected that the partition coefficient for Magnevist, whose active component is a negative ion, would be significantly lower than that of Gadovist, since cations in the disc’s interstitial fluid are depleted as a result of the fixed negative charge on the proteoglycans. However, the experimental results (Fig. 4) do not show this effect—there is essentially no difference between the Magnevist and Gadovist data. For reasons that are not obvious, Magnevist behaves as if it is uncharged. It may be conjectured that, in the environment of the disc, the negative Magnevist ion is screened by positive charges in the interstitial fluid.

For all three contrast agents, the results for partition coefficient in Fig. 4 show a similar trend of higher values in the nucleus than in the annulus. This difference between nucleus and annulus is statistically significant (*p* < 0.001, Student *t* test on paired log data for all 22 discs). On average, partition coefficients are around 80% higher in the nucleus than in the outer annulus. However, this effect is probably an artefact of the calculation method: using a single, overall value for the water fraction of the disc (see above) means that the water content of the gel-like nucleus is underestimated and the water content of the more fibrous annulus is overestimated; consequently the concentration of contrast agent is overestimated for the nucleus and underestimated for the annulus. (As a conjectural but plausible example, if the average whole-disc water fraction of 0.74 were decomposed into fractions 0.49, 0.73 and 0.87 for outer annulus, inner annulus and nucleus, respectively, the rising trend in Fig. 4 would be eliminated.)

In comparison, there have been a number of in-vivo studies on the uptake of gadopentetate dimeglumine (Magnevist) in the human intervertebral disc, using the dGEMRIC (delayed gadolinium-enhanced MRI of cartilage) technique [22], some including separate measurements of uptake in the nucleus and annulus [21, 25, 31, 32]. Vaga et al. [31] measured pre-contrast and post-contrast *T*_1_ values that were higher for the nucleus than the annulus; the contrast-related increase in relaxation rate 1/*T*_1_ was smaller for the nucleus than the annulus, suggesting a lower concentration of contrast agent in the nucleus—lower uptake of the negatively charged contrast agent may be associated with higher levels of negatively charged glycosaminoglycans in the nucleus [21]. However, Koy et al*.* [32] measured a contrast-related increase in 1/*T*_1_ that was broadly constant throughout the disc (calculated from their “pre-bedrest” data), suggesting a similar level of uptake in the nucleus and annulus.

Figure 5 shows longer time constants for manganese chloride. This is clearly not a molecular-size effect, since the manganese ion is the smallest of the three active components. (It may be that none of the solutes used in the present study is large enough to exhibit the size effects seen in previous studies [15, 16, 33]). A possible explanation is that the movement of positively charged manganese ions through the disc is hindered by electrostatic attraction to the fixed negative charges on the proteoglycans—an effect not present in the cases of Magnevist or Gadovist.

For all three contrast agents, the results for time constant in Fig. 5 suggest a similar trend of higher values in the annulus than in the nucleus. This difference between annulus and nucleus is statistically significant (*p* = 0.005, Student *t* test on paired log data for all 22 discs). On average, time constants are around 25% longer in the outer annulus than in the nucleus. This can be attributed to the disc composition: the annulus contains a tightly woven structure of collagen fibres which might be expected to hinder diffusion in the interstitial fluid, whereas the nucleus has a much looser structure of collagen fibres in a heavily hydrated gel [30].

Discs from older horses might be expected to be more fibrous than those from younger horses, and hence display longer time constants for diffusion. However, although there is a suggestion of such an age effect in the time constant data [28], not shown here, this is not statistically significant; neither is there a significant age effect for the partition coefficient. Similarly, the water fraction *f* shows little variation over the 22 samples (age range 2–30 years)—all values of *f* lie between 0.695 and 0.765.

In summary, the successful implementation of the MRI technique in the present study provides high-quality quantitative data of a type not previously available in the research literature, measured with high time resolution at multiple sites within the disc; the data correspond well to theoretical predictions. The in-vitro technique allows disc hydration to be maintained at physiological levels while providing a well-defined boundary condition for delivery of contrast agent, thus avoiding the methodological difficulties in vivo that relate to delivery via the complex microcirculation which serves the disc. Curve fitting allows values for partition coefficient and time constant to be readily determined for the diffusion of contrast agents in the disc, results which will inform future studies on the movement of contrast agents in vivo. Average time constants of around 14, 9 and 6 h were observed for manganese chloride, Magnevist and Gadovist, respectively. In terms of a model for the transport of nutrients or pharmaceuticals [34] into the disc, these figures may be considered as providing low estimates of the times involved: evaluation of Eq. 1 for *t* = *τ* and *t* = 2*τ* shows that the concentration *C* reaches only 53% of its eventual concentration *C*_*∞*_ after one time constant, and 83% after two time constants. In vivo, the significant distance between the disc surface and capillaries in the neighbouring bone may further slow these processes, as also may the presence of the disc end plate, although cyclic loading may generate convective transport to increase the speed. The observation of longer time constants and higher partition coefficients for manganese chloride, the positively charged agent, may also have implications for the delivery of nutrients or pharmaceuticals to the disc: time constants and partition coefficients for positively charged metabolites may be similarly affected; compared to a neutral drug transporter, a positively charged transporter may deliver a drug more slowly but its final concentration may be higher.
